# Genome-guided discovery of clavaic acid, a decalin-containing polyketide encoded by a LovB-like polyketide synthase

**DOI:** 10.1093/jimb/kuag022

**Published:** 2026-08-11

**Authors:** Yu Ping, Zainab Batool, Zhen Fan, Shaonan Liu, Anirudh Lahiri, Zixuan Pang, Yang Hai

**Affiliations:** Department of Chemistry and Biochemistry, University of California Santa Barbara, Santa Barbara, CA, United States; Department of Chemistry and Biochemistry, University of California Santa Barbara, Santa Barbara, CA, United States; Department of Plant and Environment Sciences, University of Copenhagen, Thorvaldsensvej 40, Frederiksberg C, Denmark; Department of Chemistry and Biochemistry, University of California Santa Barbara, Santa Barbara, CA, United States; Interdisciplinary Program in Quantitative Biosciences, University of California Santa Barbara, Santa Barbara, CA, United States; Department of Chemistry and Biochemistry, University of California Santa Barbara, Santa Barbara, CA, United States; Department of Chemistry and Biochemistry, University of California Santa Barbara, Santa Barbara, CA, United States

**Keywords:** genome mining, polyketide, LovB-like, clavaic acid, biosynthesis

## Abstract

Fungal iterative type I polyketide synthases (iPKSs) generate structurally diverse natural products. A subset of these assembly line enzymes, exemplified by LovB from *Aspergillus terreus*, contains a C-terminal condensation domain bearing a noncanonical HRxxxDG motif. While these LovB-like iPKSs are widely distributed in fungi, the majority remain uncharacterized, leaving both their associated polyketide products and the function of their unusual condensation domains largely unexplored. Here, we report the characterization of a LovB-like iPKS from *Aspergillus clavatus*. Heterologous reconstitution of this PKS system in *Aspergillus nidulans* led to the discovery of clavaic acid, a previously undescribed polyketide featuring a trimethylated *trans*-decalin core and an all-*E*-configured carboxytriene side chain. Functional analysis of the condensation domain *in vivo* demonstrated that it is essential for clavaic acid biosynthesis. Surprisingly, whereas the conserved arginine residue within the HRxxxDG motif was dispensable for product formation, the conserved aspartate residue was strictly required. These findings expand our understanding of LovB-like iPKSs and establish this enzyme family as a promising source of cryptic fungal polyketides.

**One-sentence summary** Genome-guided discovery of a *trans*-decalin-containing polyketide encoded by a LovB-like polyketide synthase in *Aspergillus clavatus*.

## Graphical Abstract

**Graphical Abstract:**
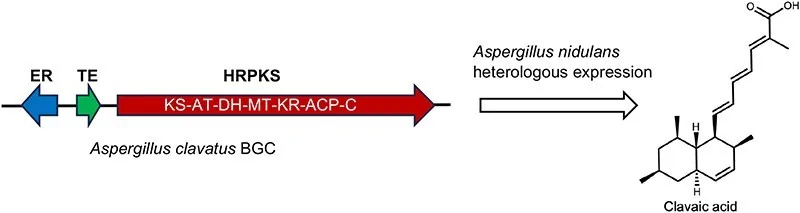

Polyketide natural products constitute a rich and chemically diverse class of secondary metabolites that serve as important pharmaceuticals, agrochemicals, and chemical probes (Klaus & Grininger, 2018; Paulsel & Williams, 2023; Stockdale et al., 2021). In fungi, the majority of polyketides are assembled by iterative type I polyketide synthases (iPKSs). These assembly-line enzymatic machines use a single set of catalytic domains in a repetitive and permutative manner to construct complex carbon skeletons (Hang et al., 2016; Herbst et al., 2018). The intricate programming of fungal iPKSs enables the formation of structurally complex natural products, but also makes it difficult to predict the structures of their products directly from gene sequences (Chooi & Tang, 2012). Consequently, continued discovery and characterization of fungal iPKSs remain essential for expanding our understanding of polyketide biosynthesis and for uncovering new natural products with potential biological activities (Mabesoone et al., 2024).

A prominent example of a fungal highly-reducing iPKS is LovB, which is responsible for assembling the nonaketide backbone of lovastatin in *Aspergillus terreus* (Ma et al., 2009). LovB operates in concert with a *trans*-acting enoyl reductase (LovC) and exhibits sophisticated programming to generate a polyene intermediate that subsequently undergoes intramolecular cyclization to form the characteristic decalin scaffold of lovastatin (Figure 1). Interestingly, LovB possesses an unusual domain architecture that distinguishes it from most fungal PKSs: in addition to canonical PKS domains, it contains a C-terminal condensation (C) domain, a domain type more commonly associated with nonribosomal peptide synthetases (NRPSs). Although the canonical C domains in NRPS systems typically catalyze amide bond formation, the role of the C domain in LovB remains unclear. Previous studies have speculated that this domain may participate in facilitating the intramolecular Diels−Alder reaction that generates the decalin ring (Ma et al., 2009). However, the precise function of the C domain remains unresolved, and it is unclear whether a conserved catalytic role is shared among the numerous LovB-like iPKSs found in fungal genomes.

**Figure 1:**
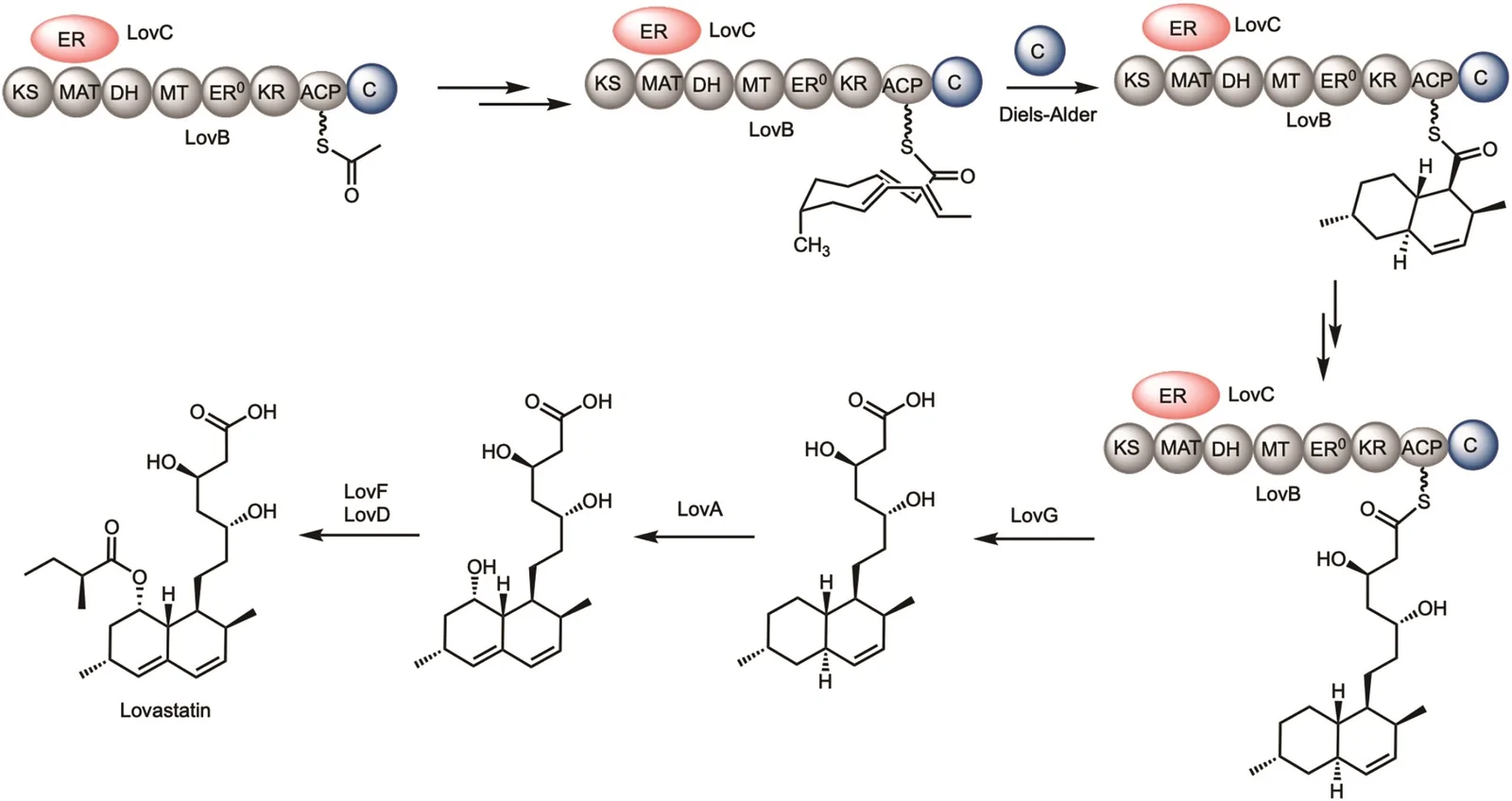
LovB, an iterative polyketide synthase involved in lovastatin biosynthesis, adopts an unusual domain architecture featuring a C-terminal condensation domain proposed to facilitate decalin-ring formation.

Bioinformatic analyses have identified a large number of fungal iPKSs featuring a C-terminal condensation domain (Hai & Tang, 2018). Like LovB, the condensation domains in many of these iPKSs contain a conserved H**R**xxxDG motif (Figure S1), rather than the canonical H**H**xxxDG motif found in NRPS condensation domains (Dekimpe & Masschelein, 2021). Although these LovB-like iPKSs are widely distributed across fungal genomes, the vast majority remain functionally uncharacterized, and their associated natural products are unknown. Collectively, these observations suggest that this LovB-like subset of fungal iPKSs represents an underexplored branch of fungal polyketide biosynthesis with significant potential to yield previously undiscovered natural products.

## Materials and methods

### General procedure for metabolite analysis

LC−MS analyses were performed on a Shimadzu 2020 LC–MS–potato dextrose agar (PDA) system with a Kinetex C18 column (100 mm × 2.1 mm, 1.7 μm) using electrospray ionization in both positive and negative modes. The mobile phases were A (water containing 0.1% formic acid) and B (acetonitrile containing 0.1% formic acid). Samples were analyzed under the following gradient program at a flow rate of 0.3 mL/min: 2% B from 0 to 1.5 min, 2 to 98% B from 1.5 to 13.5 min, 98% B from 13.5 to 16.5 min, 98 to 2% B from 16.5 to 17 min, 2% B from 17 to 20 min. NMR spectra were recorded on a Bruker Avance NEO 500 MHz spectrometer. High-resolution mass spectra (HRMS) were recorded on Q-TOF LCMS-9030 (SHIMADZU). Optical rotation was recorded on a Rudolph Research Laboratory AUTOPOL IV polarimeter.

### Strains and cultivation

*Aspergillus clavatus* NRRL1 was obtained from the ARS Culture Collection (NRRL) and cultured on PDA for 5 days at 30°C. Spores were inoculated into 10 mL potato dextrose broth and cultured at 30°C for 3 days with shaking at 250 rpm. The mycelia were collected for genomic DNA extraction using Quick-DNA^TM^ Fungal/Bacterial Miniprep kit (Zymo Research). *Escherichia coli* XL-10 was used for DNA manipulation and cultured in 2 X YT with appropriate antibiotics. *Saccharomyces cerevisiae* JHY686 was used to assemble plasmids via homologous recombination. *Aspergillus nidulans* LO7890 was used for heterologous expression (Chiang et al., 2013). For sporulation, *A. nidulans* was grown on CD medium (10 g/L glucose, 1X nitrate salts, 1 mL/L trace elements, 20 g/L agar added for plate culture) supplemented with 5 mM uracil, 10 mM uridine, 0.5 mg/L pyridoxine, and 0.25 mg/L riboflavin. For biosynthetic gene cluster (BGC) expression and compound isolation, *A. nidulans* was cultured on CD-ST medium (20 g/L starch, 20 g/L casamino acids, 1X nitrate salts, 1 mL/L trace elements, 20 g/L agar).

### Heterologous expression of *A. clavatus* genes in *A. nidulans*

All primers and plasmids used in this study are listed in Table S1 and Table S2, respectively. Gene sequences are listed in Table S3. Q5® High-fidelity DNA Polymerase and the restriction endonucleases PacI and SwaI from New England Biolabs (NEB) were used for DNA manipulation. The Frozen-EZ Yeast Transformation II Kit (Zymo Research) and the Zymoprep Yeast Plasmid Miniprep I Kit (Zymo Research) were used for plasmid manipulation in the *S. cerevisiae* JHY686 strain. The biosynthetic genes were amplified from *A. clavatus* NRRL1 genomic DNA and cloned into *A. nidulans* expression vectors pYTP, pYTR, and pYTU using the *S. crevisiae* JHY686 strain through yeast homologous recombination. The assembled plasmids were extracted from yeast using the Zymoprep Yeast Plasmid Miniprep I kit (Zymo Inc. USA) and transformed into *E. coli* XL-10 by electroporation for amplification. All plasmids were verified by enzymatic digestion check followed by whole-plasmid sequencing at Plasmidsaurus.

The protoplasts of *A. nidulans* were prepared and transformed with plasmids following a published protocol(Yee & Tang, 2022). Transformants were streaked onto CD−ST agar plates for fermentation and incubated at 28°C for 3 days. For metabolite analysis, a 1 cm^3^ piece of agar was soaked in 0.8 mL acetone and vortexed for 10 min. Insoluble materials were removed by centrifugation at 15 000 rpm for 5 min, and the supernatant was dried under vacuum. The extract was resuspended in 120 μL methanol and the resulting solution was clarified by centrifugation at 15 000 rpm for 15 min before injection into LC−MS for metabolomic analysis.

### Compound isolation and structure elucidation

A 3 L of fermentation culture of the *A. nidulans* transformant grown on CD-ST agar was incubated at 28°C for 3 days and extracted with acetone for 48 h. The crude extract was filtered, and the acetone was removed by evaporation. The aqueous solution was extracted three times with ethyl acetate containing 0.1% acetic acid. The organic layers were combined and evaporated to dryness. The crude extract was dissolved in 5% methanol aqueous solution and loaded onto a gravity C18 column for purification using stepwise gradient elution with methanol/H_2_O. Fractions containing the targeted compound were combined and the solvent was removed by rotary evaporation. The compound was further purified using a Biotage® Selekt system equipped with a C18 column using methanol and H_2_O as the mobile phase. Fractions containing the target product were combined and further purified by using an HPLC system equipped with a semi-preparative C18 column (COSMOSIL, 5C18-AR-II) with mobile phase A (H_2_O containing 0.1% formic acid) and B (acetonitrile containing 0.1% formic acid). The compound was eluted using the following program at a flow rate of 3.5 mL/min, 90% B from 0 to 20 min; 90%–98% B from 20 to 21 min; 98% B from 21 to 25 min; 98%–90% B from 25 to 26 min; 90% B from 26 to 31 min. The target compound eluted at 21 min using this method. Fractions containing the purified compound were combined, and the solvents were removed by rotary evaporation. On average, approximately 4 mg of the pure compound was obtained from a 3 L culture. All NMR spectra were shown in the supporting information and summarized in Table S4. HRMS m/z found 315.2330, calculated for C_21_H_31_O_2_ [M + H]^+^ 315.2319. [α]^20^_D_ = 23.6 (c 0.2, MeOH).

### Electronic circular dichroism (ECD) analysis

Electronic circular dichroism (ECD) spectra were recorded on a JASCO J-1500 circular dichroism spectrometer. The compound was dissolved in MeOH and transferred to a quartz cuvette with a path length of 1 cm. Spectra were collected at 25°C over a wavelength range of 400 nm–200 nm, using a scanning speed of 50 nm/min and a digital integration time (D.I.T.) of 4 s. The concentration of the sample was 63.7 μM. A MeOH solvent blank was recorded under the same conditions and subtracted from the sample spectrum.

To calculate the theoretical ECD spectra, the two enantiomers of clavic acid, the drawn absolute configuration and its mirror image (ent), were each subjected to an initial conformational search. Starting from the SMILES strings, the conformer ensembles were generated with RDKit using the ETKDGv3 distance-geometry algorithm (n = 200 initial conformers, with fixed random seed). Conformers are then optimized with the MMFF94 force field, energy-ranked, filtered within a 6 kcal/mol window, and then selected with an RMSD threshold of 0.5 Å to remove potential duplicates. The retained low-energy conformers of each enantiomer were carried forward to DFT computations. All conformers were optimized at the B3LYP-D3BJ/def2-SVP level of theory using ORCA (v6.1), with Grimme’s D3 dispersion correction (Becke–Johnson damping), the def2/J auxiliary basis, the RIJCOSX approximation, and tight SCF convergence. Solvation was modeled implicitly with the SMD continuum model using methanol as the solvent. Analytical harmonic frequency calculations were at the same level of theory on each optimized geometry to confirm that all structures were true minima (no imaginary frequencies) and to obtain Gibbs free energies at 298.15 K.

ECD spectra were then computed on the optimized geometries using time-dependent DFT at the CAM-B3LYP/def2-TZVP level, again with def2/J, RIJCOSX, and tight SCF convergence, and with the SMD methanol continuum model. The full TD-DFT formalism was used (Tamm–Dancoff approximation disabled), solving for 30 excited states per conformer. Rotatory strengths in the velocity (origin-independent) representation were used for spectral construction. Finally, for each enantiomer, conformer spectra were Boltzmann-averaged using the DFT Gibbs free energies at 298.15 K. Each electronic transition was broadened with a Gaussian line shape (σ = 0.30 eV) in the energy domain to produce a continuous Δε(λ) curve, and the weighted conformer spectra were summed to yield the final predicted ECD spectrum for each enantiomer.

## Results and discussion

**Figure 2:**
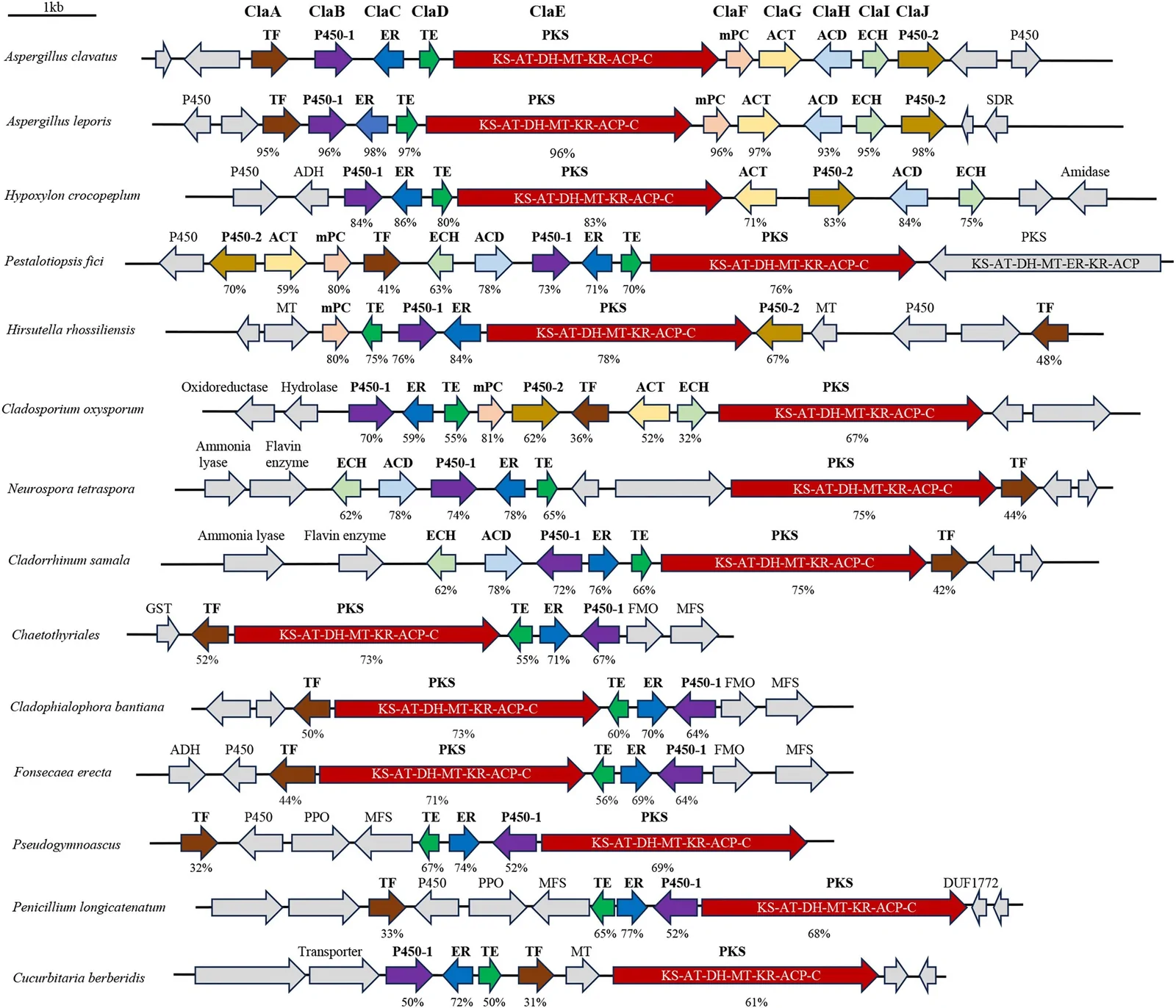
Genome mining identified a conserved polyketide biosynthetic gene cluster (BGC) encoding an uncharacterized LovB-like iterative polyketide synthase. Homologous BGCs from the same genus were identified using CAGECAT (van den Belt et al., 2023) and are shown in Figure S1. The percent amino acid sequence identities (relative to the *A. clavatus* cluster) for conserved biosynthetic genes are indicated. Gene annotation abbreviations: TF, transcription factor; ER, enoyl reductase; TE, thioesterase; PKS, polyketide synthase; mPC, mitochondrial phosphate carrier protein; ACT, acyltransferase; ACD, acyl-CoA dehydrogenase; ECH, enoyl-CoA hydratase; SDR, short-chain dehydrogenase/reductase; MT, methyltransferase; GST, glutathione S-transferase; FMO, flavin monooxygenase; MFS, major facilitator superfamily transporter; ADH, alcohol dehydrogenase; PPO, protoporphyrinogen oxidase.

In the course of analyzing genome neighborhoods of LovB-like iPKSs in publicly available databases, we identified a highly conserved BGC, which encodes a LovB-like highly-reducing iterative polyketide synthase (Figures 2 and S2). In addition to the PKS, this BGC also encodes a *trans*-acting ER, a thioesterase (TE), a cytochrome P450 monooxygenase (P450), and a transcription factor (TF). Remarkably, this BGC is conserved across diverse fungal lineages and is found in multiple fungal orders, suggesting a potentially widespread and functionally important biosynthetic pathway. In some fungal species, such as *Aspergillus clavatus*, and *Pestalotiopsis fici*, this core BGC is further expanded to include an additional P450, a mitochondrial phosphate carrier protein (mPC), an acyltransferase (ACT), an acyl-CoA dehydrogenase (ACD), and an enoyl-CoA hydratase (ECH). Given that tailoring enzymes can contribute significantly to the structural complexity and chemical diversity of the final polyketide product, we chose to characterize the BGC from *A. clavatus* (hereafter named as *cla*, Figure 2), which appears to encode the most extensive set of tailoring genes.

**Figure 3:**
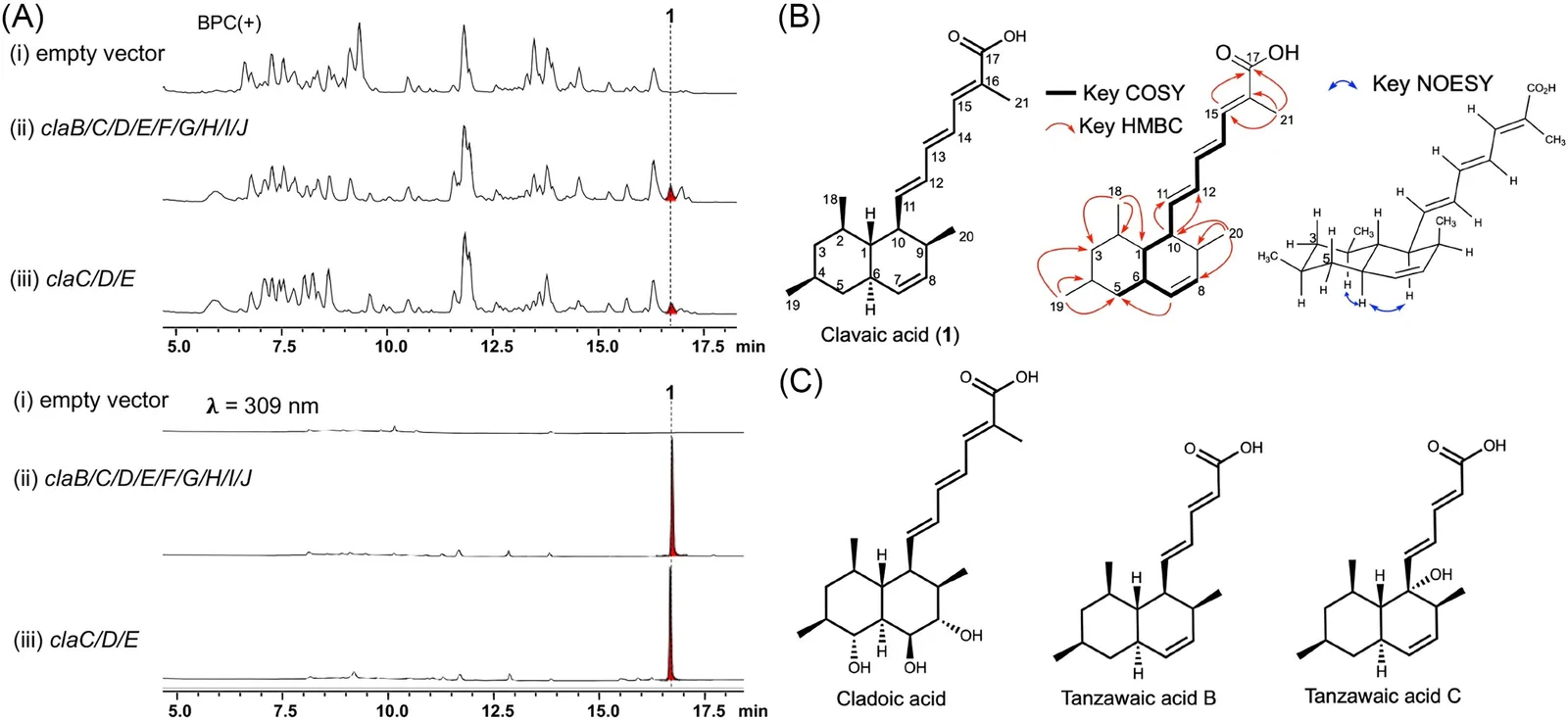
Heterologous expression of the *A. clavatus* PKS gene cluster in *A. nidulans*. (A) LC-PDA-MS analysis of metabolites extracted from *A. nidulans* transformants. (B) Structure of clavaic acid **1** and its relative configuration determination by 2D-NMR. (C) Related polyketide natural products.

To elucidate the metabolite encoded by this BGC, we introduced the complete set of biosynthetic genes (*claB*-*J*), except the transcription factor *claA*, into *Aspergillus nidulans* LO7890 strain, an engineered fungal host developed by the Oakley group in which seven endogenous BGCs have been deleted, resulting in a reduced secondary metabolite background that facilitates detection of heterologously produced compounds (Chiang et al., 2022, 2013). The resulting *A. nidulans* transformant yielded a new metabolite (λ_max_ = 309 nm), designated compound **1**, that was absent in the empty-vector control strain (Figure 3A). However, reconstitution of the minimal gene set (*claC/D/E*) in *A. nidulans* also led to the production of compound **1**, suggesting **1** may be the direct product of the PKS assembly line.

Compound **1** was obtained from large-scale cultivation and purified by reversed-phase chromatography. Its molecular formula was determined as C_21_H_30_O_2_ by positive-mode HR-ESI-TOF-MS, corresponding to seven degrees of unsaturation. The ^1^H NMR spectrum showed four methyl groups (*δ*_H_ 1.93, 0.97, 0.96, 0.90) and seven olefinic protons, while the ^13^C NMR spectrum displayed 21 carbon resonances, including one carboxylic acid carbon, eight olefinic carbons, and multiple aliphatic carbons (Table S4). The carboxylic acid carbon and four double bonds accounted for five degrees of unsaturation, leaving two additional degrees of unsaturation attributable to a bicyclic ring system.

The planar structure of **1** was established by integrated analysis of the COSY, HSQC, and HMBC spectra (Figures S6–S8). HMBC correlations from H_3_-18 to C-1/C-2/C-3 and from H_3_-19 to C-3/C-4/C-5 established a C-1–C-5 fragment bearing two methyl substituents at C-2 and C-4, whereas correlations from H_3_-20 to C-8/C-9/C-10 defined a C-8–C-10 fragment bearing a methyl substituent at C-9. These fragments were connected by COSY correlations from H-5 to H-8, together with additional correlations involving H-6, H-1, and H-10, thereby establishing the trimethyl-substituted decalin core. The terminal α-methyl carboxylic acid moiety was defined by HMBC correlations from H_3_-21 to C-15/C-16/C-17 and from H-15 to C-17, and was connected to the decalin core by sequential COSY correlations from H-10 to H-15, together with HMBC correlations from H-10 to C-11 and C-12. Collectively, these COSY and HMBC correlations established the planar structure of **1** as a trimethylated decalin polyketide bearing a conjugated triene side chain terminating in an α-methyl carboxylic acid moiety.

The relative configuration of the decalin ring system was assigned by coupling-constant analysis and key ^1^H–^1^H NOESY correlations (Figure 3B and Table S4). For clarity, the face containing H-2, H-4, H-6, and H-10 was designated as the α-face. Small coupling constants of *J*_2,3a_ = 3.0 Hz, *J*_3a,4_ = 3.0 Hz, *J*_4,5a_ = 3.0 Hz, and *J*_5a,6_ = 3.0 Hz, together with NOESY correlations of H-6 with H-2 and H-10, placed H-2, H-4, H-6, and H-10 on the same face. In contrast, the large coupling constant between H-1 and H-6 (*J*_1,6_ = 10.2 or 12.7 Hz) placed H-1 on the opposite face. The coupling constants associated with H-10 were assigned as *J*_1,10_ = 9.8 Hz and *J*_9,10_ = 5.5 Hz, supporting the relative orientation of H-9 and H-10 within the trans-fused decalin system. The conjugated double bonds in the side chain were assigned as all-E based on large olefinic coupling constants (*J* ≈ 14–15 Hz). Collectively, these data established **1** as a highly reduced nonaketide featuring a trimethylated unsaturated *trans*-decalin core and an all-E conjugated triene side chain terminating in an α-methyl carboxylic acid moiety. The absolute configuration of **1** was determined by comparison of calculated and experimental ECD spectra (Figure S4) for both possible enantiomers of **1**.

A structure-based search using SciFinder confirmed that compound **1** is a previously undescribed natural product, and we named it clavaic acid based on its biosynthetic gene origin. Its configuration matches the carbon skeleton of cladoic acid (Figure 3C), a recently discovered anti-*Trypanosoma cruzi* polyketide isolated from *Cladosporium* sp. TP-F2020 (Rana et al., 2024). The scaffold of clavaic acid also resembles that of hynapenes (Tabata et al., 1993) and tanzawaic acids (Kuramoto et al., 2004), both of which possess a similar trimethylated *trans*-decalin core but harbor a carboxy diene side chain instead of a triene (Figure 3C). The presence of a highly oxygenated decalin ring in cladoic acid suggests that clavaic acid may undergo further oxidative tailoring in its native biosynthetic pathway, presumably catalyzed by the cytochrome P450s encoded within the gene cluster. Accordingly, multiple strategies were employed to enhance ClaB and ClaJ expression, including promoter replacement and the introduction of intron-free versions, while all other genes were expressed in their native forms. Despite confirmation of claB and claJ expression in *A. nidulans* by reverse transcription PCR, no metabolites beyond **1** were detected. We speculate that additional factors may be required for the downstream oxidative transformations, such as accessory proteins, redox partners, or host-specific environments that are currently absent in the heterologous host.

**Figure 4:**
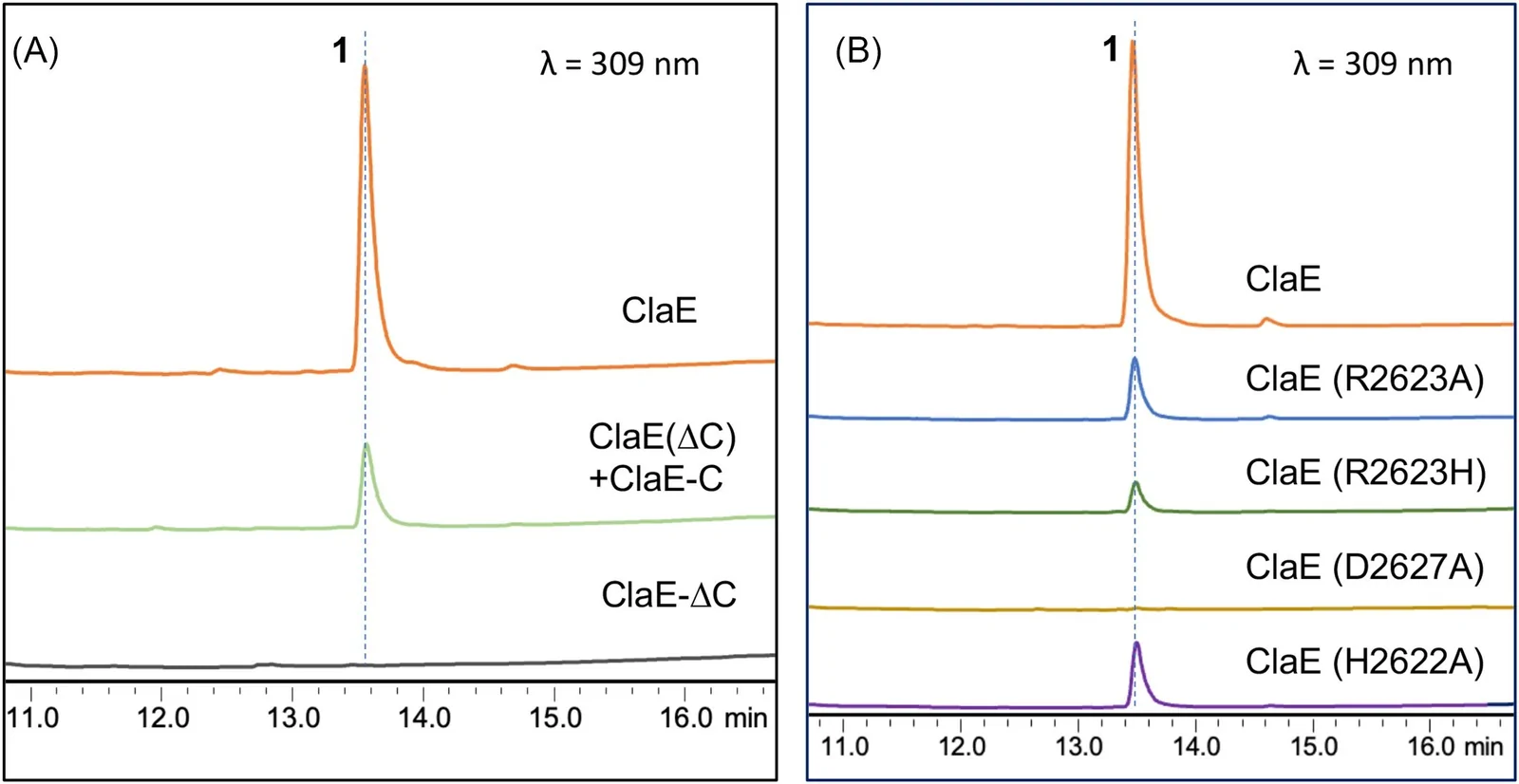
Probing the function of the condensation domain via heterologous expression. (A) Activity of PKS C-domain truncation variant. (B) Activity of PKS C-domain active site mutants.

To investigate the necessity of the C-terminal condensation (C)-domain of LovB-like iPKS ClaE in the biosynthesis of clavaic acid, a truncated ClaE lacking the C domain (ClaE-ΔC) was constructed. Expression of ClaE-ΔC with TE and ER in *A. nidulans* failed to produce compound **1** (Figure 4A), indicating that the C domain is essential for product formation. Notably, co-expression of the standalone C domain (ClaE-C) with ClaE-ΔC restored production of compound **1**, demonstrating that the C domain can function *in trans*, a behavior reminiscent of the condensation domain in LovB (Ma et al., 2009).

To further probe the catalytic role of this C domain, we focused on the HR^2623^xxxDG motif in ClaE, which is characteristic of all LovB-like iPKSs. This motif represents a natural variant of the canonical H**H**xxxDG motif found in NRPS condensation domains, where the corresponding histidine residue plays a critical catalytic role in amide bond formation ((Heberlig et al., 2025; Pistofidis et al., 2025). We therefore hypothesized that this position may retain catalytic functionality while adapting to a distinct, nonamide-bond forming activity. To test this possibility, R2623 was mutated to either alanine or histidine (Figure 4B). In contrast to canonical NRPS C domains, where mutation of the second histidine abolishes activity (Bloudoff & Schmeing, 2017), both R2623A and R2623H variants retained the ability to produce compound **1**, albeit at reduced titers. These results indicate that, although the C domain is critical for LovB-like iPKS function, the arginine residue in the HRxxxDG motif is not essential for catalysis. We next examined the remaining conserved residues within the HRxxxDG motif by mutating H2622 and D2627 (Figure 4B). Whereas mutation of H2622 only partially impaired production, substitution of D2627 completely abolished the formation of **1**. Together, these results indicate that the catalytic role of the LovB-like iPKS C domain is mechanistically distinct from canonical C domains and reveal an unexpected requirement for D2627. Elucidating the precise function of this domain and the mechanistic contribution of D2627 will require future biochemical and structural studies.

**Figure 5:**
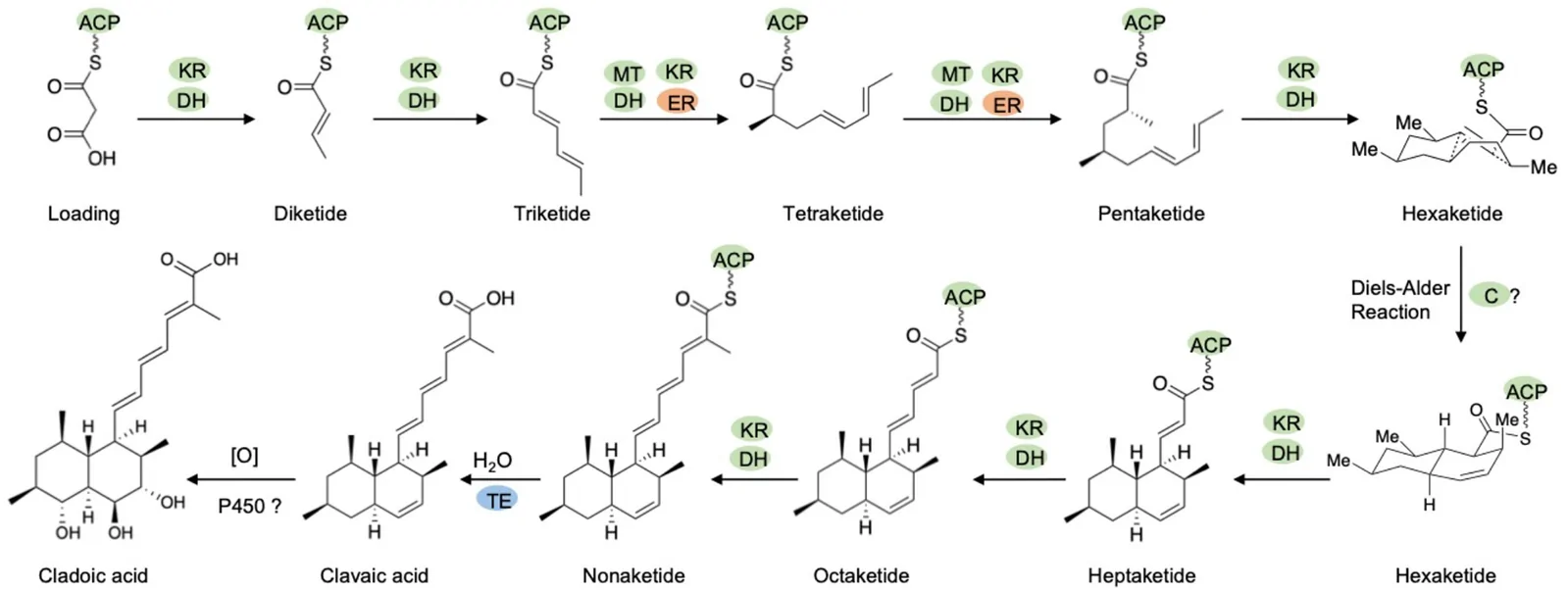
Proposed biosynthetic pathway of clavaic acid.

Taken together, we propose a plausible biosynthetic mechanism for clavaic acid (Figure 5). Similar to lovastatin biosynthesis, the decalin ring in clavaic acid is likely formed through an *endo*-selective intramolecular Diels–Alder reaction of a polyene intermediate at the hexaketide stage. The strict requirement for the C domain and the conserved D2627 residue suggests that this domain participates in a key step during product assembly. However, its precise function remains unresolved. Furthermore, unlike LovB, for which a stereoselective Diels–Alderase activity was demonstrated using a synthetic substrate analogue (Auclair et al., 2000), it remains unclear whether decalin ring formation in clavaic acid biosynthesis requires enzymatic catalysis. In the LovB system, enzyme catalysis is thought to be necessary to overcome an unfavorable kinetic barrier imposed by an axial methyl group on the substrate. In contrast, the equatorial methyl groups in the proposed clavaic acid precursor may allow the intramolecular Diels–Alder reaction to proceed diastereoselectively without enzymatic assistance (Figure 5).

A recent genetic study identified a LovB-like iPKS responsible for the biosynthesis of tanzawaic acids in *Penicillium steckii* (Bernal et al., 2023). In light of the structural similarities between clavaic acid and tanzawaic acids, it is plausible that the C-terminal condensation domain plays a unified role in these systems. Furthermore, as LovB also produces a *trans*-decalin-containing polyketide, this observation raises the possibility that the LovB-like protein family may be generally programmed to generate decalin scaffolds (Jamieson et al., 2019). If so, it will be important to determine whether *trans*-configuration is a general feature and whether this stereocontrol can be reprogrammed.

## Conclusion

In summary, we identified and characterized a LovB-like iPKS system from *Aspergillus clavatus*, and established its product, clavaic acid, as a previously undescribed *trans*-decalin-containing polyketide. We demonstrated that the C-terminal condensation domain is essential for clavaic acid biosynthesis and can function *in trans*. Mutagenesis of the conserved HRxxxDG motif further revealed an unexpected requirement for D2627, providing a foundation for future studies aimed at elucidating the catalytic role of the unusual C domain harboring this motif. Our work highlights the power of a genome-guided approach and establishes LovB-like iPKSs as an underexplored source for uncovering new polyketide natural products.

## Supplementary Material

kuag022_Supplemental_File

## Data Availability

The Supporting Information is available online.
