# Supplementary material for: Genome-guided discovery of clavaic acid, a decalin-containing polyketide encoded by a LovB-like polyketide synthase
Source: J Ind Microbiol Biotechnol. 2026 Aug 11;53:kuag022. doi: 10.1093/jimb/kuag022 (PMC13492342; doi:10.1093/jimb/kuag022)
Supplement: Supplementary file 1 [file kuag022_supplemental_file.docx]

**Supporting Information**

**Genome-guided discovery of clavaic acid, a decalin-containing polyketide encoded by a LovB-like polyketide synthase**

Yu Ping,^1#^ Zainab Batool,^1#^ Zhen Fan,^2^ Shaonan Liu,^1^ Anirudh Lahiri,^3^ Zixuan Pang,^1^ and Yang Hai^1^*

^1^Department of Chemistry and Biochemistry, University of California Santa Barbara, Santa Barbara, California, 93106, USA.

^2^Department of Plant and Environment Sciences, University of Copenhagen, Frederiksberg C 1871, Denmark

^3^Department of Chemistry and Biochemistry, University of California Santa Barbara, Santa Barbara, California, 93106, USA.

^4^Interdisciplinary Program in Quantitative Biosciences, University of California Santa Barbara, Santa Barbara, California 93106, United States

^#^ These authors contributed equally

**Correspondence and requests for materials should be addressed to Y.H. (*[*hai@chem.ucsb.edu*](mailto:hai@chem.ucsb.edu)*)*

**Table of Contents**

[1 Supplementary Tables	3](#_Toc232411557)

[Table S1. Primers used in this study.	3](#_Toc232411558)

[Table S2. Plasmids used in this study.	5](#_Toc232411559)

[Table S3. Sequences of the three functionally characterized genes in this study.	7](#_Toc232411560)

[Table S4. Structural characterization of clavaic acid (1).	10](#_Toc232411561)

[2 Supplementary Figures	11](#_Toc232411562)

[Figure S1. Multiple sequence alignment indicates the conserved HRxxxDG motif found in the condensation domain of LovB-like iPKS.	11](#_Toc232411563)

[Figure S2. Comparison of homologous gene clusters from representative species.	12](#_Toc232411564)

[Figure S3. UV-Vis absorption spectrum and HR-MS spectrum of clavaic acid (1).	13](#_Toc232411565)

[Figure S4. Experimental and calculated ECD spectra of clavaic acid (1).	14](#_Toc232411566)

[Figure S5. ^1^H NMR (500 MHz) of clavaic acid (1) in CD_3_OD.	15](#_Toc232411567)

[Figure S6. ^13^C NMR (125 MHz) of clavaic acid (1) in CD_3_OD.	16](#_Toc232411568)

[Figure S7. ^1^H-^13^C HSQC NMR (500 MHz) of clavaic acid (1) in CD_3_OD.	17](#_Toc232411569)

[Figure S8. ^1^H-^1^H COSY (500 MHz) NMR of clavaic acid (1) in CD_3_OD.	18](#_Toc232411570)

[Figure S9. ^1^H-^13^C HMBC (500 MHz) of clavaic acid (1) in CD_3_OD.	19](#_Toc232411571)

[Figure S10. ^1^H-^1^H NOESY of clavaic acid (1) in CD_3_OD.	20](#_Toc232411572)

# 1 Supplementary Tables

## Table S1. Primers used in this study.

| Primers | Sequence (5’-3’) |
| --- | --- |
| pYTU_PKS_F1 | AGCTTCATCCCCAGCATCATTACACCTCAGCATTAATTAAATGACCCAATCTGAAGCCCG |
| pYTU_PKS_R1 | TTTTAGCAGAGATCGCACATTTGCC |
| pYTU_PKS_F2 | GCCGTCCTCCAAGCGAGCAAGACTT |
| pYTU_PKS_R2 | GTCAGATGTCCTAGAGCATTTCCGAG |
| pYTU_amyB_F | CAGTTTACTAGGGTCTCGGAAATGCTCTAGGACATCTGACGATTAAAGGTGCCGAACGAG |
| pYTU_amyB_R | TTGTTGGAGGCACAAGGGCCGCAGTATCAACCGATCCCATAAATGCCTTCTGTGGGGTTT |
| pYTU_ER_F | ATGGGATCGGTTGATACTGCGG |
| pYTU_ER_R | TACTAGAATATCTGTTGCTGTTCCT |
| pYTU_gpdA_F | CTGTCAGTCTATTTCAGGAACAGCAACAGATATTCTAGTAACTCCGGTGAATTGATTTGG |
| pYTU_gpdA_R | CATGGAAGCAAAGAAGGGCTTTGGACGGTACCCTAGACATTGTTTAGATGTGTCTATGTG |
| pYTU_TE_F | ATGTCTAGGGTACCGTCCAAAG |
| pYTU_TE_R | ACAGTGGAGGACATACCCGTAATTTTCTGGGCATTTAAATCCAAAATCTCACCGGGCCTT |
| pYTP_P450-1_F | TCTCTGAACAATAAACCCCACAGAAGGCATTTTTAATTAAATGATAGAAAAAGTCTATGC |
| pYTP_P450-1_R | TGGTGGATGATGGTAAACTG |
| pYTP_glaA_F | ATCCGCACGGGATGATGTCGCAGTTTACCATCATCCACCACCTGATCTTCCGAACTGGTC |
| pYTP_glaA_R | CAGGGACGGTCGGGGCTACAGCAGCGGGAGACGCAGCCATTGCTGAGGTGTAATGATGCT |
| pYTP_mPC_F | ATGGCTGCGTCTCCCGCTGC |
| pYTP_mPC_R | GCCCGTTCTCTTGCTGACGC |
| pYTP_gpdA_F | TATTGAGATGCCAATCGAGAGCGTCAGCAAGAGAACGGGCACTCCGGTGAATTGATTTGG |
| pYTP_gpdA_R | AAGCTGTCAGGAAGATTGGTCCAAGATTCTCGCGGAACATTGTTTAGATGTGTCTATGTG |
| pYTP_P450-2_F | ATGTTCCGCGAGAATCTTGG |
| pYTP_p450-2_R | GTGATGAGACCCAACAACCATGATACCAGGGGATTTAAATACGGCCTGAAACAAGCACCT |
| pYTR_ACT_F | CCATTACCCCGCCACATAGACACATCTAAACATTAATTAAATGGCTCGACGATGGGAGGA |
| pYTR_ACT_R | GCGTCGAAAGAATAAGTCGGG |
| pYTR_amyB_F | ATGATGAGCACTCGAAGATCCCGACTTATTCTTTCGACGCGATTAAAGGTGCCGAACGAG |
| pYTR_amyB_R | GCTCCGCGAAAGGGGTGCTGGATCCAAAAAACTCGGCCATAAATGCCTTCTGTGGGGTTT |
| pYTR_ACD_F | ATGGCCGAGTTTTTTGGATCC |
| pYTR_ACD_R | CTGATGCGCACGGCAAGGAA |
| pYTR_glaA_F | GCAATAACGACCTCAATCACTTCCTTGCCGTGCGCATCAGCCTGATCTTCCGAACTGGTC |
| pYTR_glaA_R | GCTCGTCATCGCGAGTGCTGAAAAGGTTCAAATAAGACATTGCTGAGGTGTAATGATGCT |
| pYTR_ECH_F | ATGTCTTATTTGAACCTTTTCAGC |
| pYTR_ECH_R | GCTAAAGGGTATCATCGAAAGGGAGTCATCCAATTTAAATTTCATTACCGTGACAATGGC |
| pYTP_P450_1_F1 | TCTCTGAACAATAAACCCCACAGAAGGCATTTTTAATTAAATGCTTGGGGTCTTGCTCCT |
| pYTP_P450_1_R1  pYTU_PKS-only_F | GTGATGAGACCCAACAACCATGATACCAGGGGATTTAAATTGGTGGATGATGGTAAACTG  CATCCCCAGCATCATTACACCTCAGCATTAATTAAATGACCCAATCGAAGCCCGTCGC |
| pYTU_PKS-only-R1  pYTU_PKS_F2  pYTU_PKS_R2  pYTU_RtoH-mutant_F  pYTU_RtoH-mutant_R  pYTU_RtoA-mutant_F  pYTU_RtoA-mutant_R  pYTU_ΔC-domain_R  pYTR-TE_F  pYTR-TE_R  pYTP-ER_F  pYTP-ER_R  pYTP-ER+CON_F  pYTP-ER+CON_R  pYTP-ER+CON_R1 | TTTTAGCAGAGATCGCACATTTGCC  GCCGTCCTCCAAGCGAGCAAGACTT  GGAGGACATACCCGTAATTTTCTGGGCATTTAAATGTCAGATGTCCTAGAGCATTTCCGA  TGCTTACCATCATCTTGTCGGCGATGG  CGCCGACAAGATGATGGTAAGCAATGATAAGAAGGTG  TGCTTACCATGCTCTTGTCGGCGATGG  CGCCGACAAGAGCATGGTAAGCAATGATAAGAAGGTG  GGACATACCCGTAATTTTCTGGGCATTTAAATTTATTCAAGTGTCAAGCGGGAGACTGCC  ACCCCGCCACATAGACACATCTAAACATTAATTAAATGTCTAGGGTACCGTCCAAAGCC AGGGTATCATCGAAAGGGAGTCATCCAATTTAAATCCAAAATCTCACCGGGCCTTTAGGG  TGAACAATAAACCCCACAGAAGGCATTTTTAATTAAATGGGATCGGTTGATACTGC  GAGACCCAACAACCATGATACCAGGGGATTTAAATTACTAGAATATCTGTTGCTGTTCCT  CTGAGCTTCATCCCCAGCATCATTACACCTCAGCAATGGCTCATTCTACGTCGTCGTCGGAA  GAGACCCAACAACCATGATACCAGGGGATTTAAATGTCAGATGTCCTAGAGCATTTCCGA  TAGGTCGCCAGGTACGACCAGTTCGGAAGATCAGGTACTAGAATATCTGTTGCTGTTCCT |

## Table S2. Plasmids used in this study.

| Plasmid | | | Plasmid map |
| --- | --- | --- | --- |
| pYTU-PKS-ER-TE |  | | |
| pYTP-P450_1 | |  | |
| pYTP-P450_1-mPC-P450_2 | | |  |
| pYTR-ACT-ACD-ECH  pYTU-PKSΔC  pYTR-TE  pYTP-ER  pYTU-PKS-C | | |         |
| pYTP-ER-C | | |  |
|  | | |  |

## Table S3. Sequences of the three functionally characterized genes in this study.

| gene | | | cDNA sequence |
| --- | --- | --- | --- |
| *claE* | | ATGACCCAATCTGAAGCCCGTCGCCATGAGCCCATTGCAATCGTTGGCAGCGGTTGCCGCTTCCCCGGCAGTGCCAGCAGTCCTTCTAAGCTCTGGGAACTTCTGAAAGCCCCCAGGGACCTGGTGAGGAAAGTCCCCAAGGAGAGATTCAACGTGGACGCTTTCTATCATCCCGATGGAGCCCACCATGGTCGCACGAACGCACGATTCGCCTACTTCCTTGACGAAGACCCATACACCTTTGACCCAGCCTTCTTCAATATTCCACCCAACGAAGTCGACACAATCGATCCCCAACAGCGTCTTCTTCTAGAAACTGTATACGAAAGTCTCTCGGCCGCTGGTCTCAAGATAGAAGACCTTCGAGGTTCTCCCACCGCCGTCTACGTTGGTATGATGCAACGTGATTTCCTCGACAACCAGAACTACGATCTTGATGCCCTGAACATCTATGCCGCTACCGGCACATCTGCATCCATTCTATCCAACAGAGTCTCCTACGTTTTCGACTGGCACGGTCCGAGTATGACCTTTGATACCGCCTGCAGCTCCTCCCTCGTTGCTGTTCACCATGCCATCGAGCAACTTCGAACGGGCTCAAGTCAGGTCGCCATTGCTGCCGGCTCGAACCTGATCCTGGGTCCAGTTCCTTTTATCTCAGCCAGTAAATTGAACATGTTCTCACCCACTGGCCGTTCTCGAATGTGGGATGCCGCAGCGGATGGTTATGCCAGAGGCGAAGGCGTTGCCGCTGTCGTCCTGAAAACCCTCAGTCAGGCGATCGCAGATGGTGACAGCATAGAGTGTATCATTCGTGAGAGCGGCATCAACCAGGACGGCAAAACTGCGGGAATTACAATGCCGAACCATGCGGCACAGGAAGCTCTCATTCGCGAAGTCTACAAGCGAGCCGGCCTCGATGTGACAAAGCCCGAAGACCGCTGCCAATACTTCGAAGCGCACGGTACAGGTACTCCGGCAGGAGATCCTCAAGAAGCCCAAGCCATCTCTTCTGCTTTCTTCGGGGAGAGGCAGCGTGCTGTCGACGAAGACCCTCTGTACGTGGGTAGTATTAAGACGATCATCGGTCATACTGAGGGTACTGCTGGTATTGCCGGCCTGATCAAAGCTTCGCAGGCCATTCAGCATGGTCTACTACCGCCAAATATGCTATTCAATGACCTAAGCCCGAGAGTGGCTCCATTCTACTCGGATCTCCAGCTTGTCACGAAGCTCCAGGCCTGGCCCGAGCTCAAGTCTAACCAACCACGACGGGTGAGTGTTAACTCGTTCGGATTCGGCGGTACCAACGCGCACATAATTGTGGAGTCCTATATTCCCGCCTGGAAACCAGCAATAAATGAGCTTTCAGCCCCTTGCCTTGCCCCGATCACGCTCTCCGCCAATTCAGAAGCCTCGCTGAAGGCCACCATGGAGGAGCTGAGTCAATATATCAAAAGCCAGCCCAGCCTACGGCTACAAGACCTCGCCTGGACACTGCTGAAAAAGCGATCGAACCTCCAAGTGCGCCATGCTATCCCAGCCAAGACGGTGCAAACTCTCTGCGAGGCTCTCGAGCGTGATGCCGCTTTGGTGCAAGGGAAGCAAACCATCGCAACTGTCTCGGATGTCAAGATCAAACCCGAAGTGCTTGGTATCTTCACCGGGCAGGGCGCCCAGTGGCCAACCATGGGCAAGGCTCTTATCTCGGAAGTACCGTACGCCCGCGATATCATCTTGGAGCTTGACCAATCTTTGCAGGACTTGCCAAGGGAATACCGACCCACGTGGAAGCTCATGGACCAGCTCCTTCTCGAGGGGGCGGACTCTAATGTACATGACGCCACGTTTTCGCAGCCTCTCTGCTGTGCCGTCCAGATCATGCTCACAAAGCTTCTTGAAGCTTCCGGAGTGGGATTCAAGGTTGCCGTCGGCCACAGCTCGGGAGAGATAGCTTGCGCATTTGCCGCCGGCTTCATATCGGCGTCCCAAGCAATCCGTATTGCATACCTTCGCGGTCTCACTTCGAGGCATGCAGGTAGCCTGAACGGCATGGAGGGCGCCATGATGGCTGCAGGCACCTCCTTTGATGATGCAACTGAATTATGCGCCCTCGAGGCTTTTGAGGGTAGAATAACCGTTGCAGCAAGCAATGCACCGGAAAGTGTTACCCTCTCCGGAGACAAAGATGCCATCCTCGAGGCTCAGGAGATCCTCAACGACGAGTCAAGATTCAACCGGCTCCTGAAGGTTGACAAGGCGTACCACTCTCACCATATGCGTCCTTGCGCCGAGCCCTATGTTGCAGCTCTGAAAGCTTGTGGCTGCGATAAGTTCAGTATGGATGTCGCACCCACGGCCACCTGGATCTCGTCGGTCTATGAAGGGAAGATCATGAAGCCCGAGGACCTCAAGGCTGAGTATTGGAGTGATAATCTGCTTTCTCCCGTTCTCTTCTCCTATGCTGTCGAACAAGCGCTGGTGAAGCACATGCCGCTCGATGTTTGCATCGAAGTTGGAGCTCATCCGGCTCTGAAGAATCCTAGTCTCCAGACCATTGAAAATTGCGCCGGCGCTCCGCTCCCATACGTGGGCTGCATGGAGCGGAATAAGTATGACGTTGAAGCCTTCTCTTCGTGCCTAGGCTATATATGGTCGCGCTTTGGCTCAGCTGCTATCGACGTCGATAGCCTGTACAGTAGCCTCTCATTGGATGCCAAATCCAAAGACTTGTCCAAAGGACTTCCATGCTATTCTTGGGACCATCACCGCTCGTACAGAAAGGAGTCACGCGCACTTCGCAGTTGGCTTGGAGCAGAAAGTCCTCATCTTCTGCTCGGCAAGCAACTCGTCCACAGCAGTCCTTCATCGATCCTGTGGCAGAACTTTATCCGGCAGCGTGATATCGAATGGCTCGATGGCCACTCCTTACAGGGACAGACTGTGTTTCCAGGCGCAGGATACGTTGTCATGGCCATGGAGGCTGCTATGAAGCTGGCCGGTGATCAAGAGGTTCGGCTTTTGGAAGTTCTGAACCTCTCGATCGACAAGGCCGTGACCTTTGAGGATGAAAACAGCATGGTCGAGATCAACCTCAGTCTAGACATCGACCCATCGCAGACCAAAGATACATACGCCGTATACAGCTTCGTTATCAACTCTTGCCTCGCCAGAGAAACTGGTCTTACTCGCTCAGCGGCTGGTACGGTCGCCGTCACTTACGGACCAGGAGCACCTGAGACCCTTCCACCCCCTCAAGGCGAGCCGCCTCACCTAAACAATGTTAGCATCGACAGATTTTACAACATGCTCGGTGAAATCGGGTACGGCTACACCAAGCAGTTCCGAGGAGTCAGTACCCTGAGACGCGGCGACAGCAAGGCTTGTGGCTCCATCACCTTCCACCGGCTGGAAGACAATCACCGCAACATGGTCCTACATCCTGCTACTCTCGACGTTGCCTTTCAAAGCTTCATCGGAGCGTACACTGCTCCCGGAGACAGACGCCTGCGATCCCTTCTCGTGCCTACTGGGATTGATCGCATTGCTCTGAACCCGTGGGTCGCTGATCGTATTCACCCGCTGTCCAGCCAGGTGGAGTTCATCTCAACCAGTGCGGCAAGTGTCGGAAATACCGTGGCAGGAGACATTGAAGTTGTTGATCCTGAGACCGAAGCCACCATGATCCACATCGAGGGCCTCAGTTTCAAGCCATTTTCTCCGCTCTCGGCTGCTGATGACCATGAAATGTTCTCCAAGTGGAGCTGGGGTCCAATCAACCCAGATCCCTTGCTCGATGACAGCAAATACCACGCCACGGAGCAGGACAAGAAGGACGTTGCAGTCATTGAGCGAATTACGTACTGGTACATCAAAGCGTTTCTCGCAAACCTCACGGCTGAGGACCGCGAGAAGGCAGCCTTTCACTTTACGAAGCATATCCAGTGGTGCGAGTATATCATCGTGGAGACTGAGGCGGGCCGTAATGTGTGGTACGAGCCGGGCTGGGACAAGGACACGAGAGCTGATATTGAAGAAATGATTCGACAAAATGTGGCCCATCCCTTCGTACGCCTCATTCAGAGAGTCGGTGAAGGAGCCTTGGACACACTTCGCAACAATACGAACGCCTTTGACCTGATGGACCACGATGGCTTGCTCACCGAATTCTACGGCGGCACCGTGAGCTACGGGCATTCATACCACTACTATCAAAGACTCCTTGGGCAGATCAATCACCGGGATCAAAACCTGAATGTTCTCGAAATTGGCGCTGGTACTGGGGGTGCCACACGGTATTTCTTGAACCACCAACAGATCTCGTTCAATAGCTACACCTTCACCGATATCTCCAGCGCGTTTTTCGAGCAAGCGGCCAAGGAGTTTGAGCGGCATGCCGACAAGATGGATTTCCGTCCGCTCGATATTCGCCGTCCTCCAAGCGAGCAAGACTTCAAGCCCAATTCGTACGACTTGATCATTGCTTCCAACGTGCTCCATGCCACGCCTAGGCTCGAAGAGACTCTGGCAAATGTGCGATCTCTGCTTAAACCTGGGGGTCGATTGGTTGTCATTGAAGTTGCACACCGTGAGCACACCCGTATTGGATTCATCTTCGGTTTGTTCCCCGATTGGTGGGCTGGTCATGACGAAGGGCGTGTTCTTGAACCCTTTATCTCATATGATCAGTGGGACACACTGCTGAGGAAGACTGGGTTTTCGGGTATCGACAGCCGCACTCTTGATCCGGACAGCAGGGTCTTCCCCAACGGCGTGTTCGCTACTCACGCGGTCAACGATCTAGTACAAAGGCTGGACTCGCCACTGACTGCCCCCGTCAAGGAGTCCTACGCGCCACTAGTTGTTATTGGGGGTTCCTCACCGAAGACGAGGGCGCTCTTGGATAAGCTTCCAGCCATCCTGCCAGCGCGCAAATGGGAGACTGTCCCCACCATCCGAGAGATCATCGACTTTGACTACCAGCCAGGGTCCACATTTGTTGTGCTATCTGAACTGGACCAGCATACGTTTGCTGGGTTCGATGATGAGCAACTTGATGCCTTGCAAACCATCTTCAACGCCGCCAACCATGTCTTGTGGGTGATGGAAGATGCCTGGGTTGAAAATCCGCAGCAAGGCATGACCATCGGCCTCCTTCGCACTCTCCGTATGGAATATCCGGAAATCCACATACAGGTGCTGGATGTGCAGAAGGCTGAAAATCTGAGACCTGAGTTGCTGGTTGAGACCATTCTGCGGTTGGAAGATGGTACTGACTGGCAGGAGAGCGGTATTTTGTGGACACAGGAGCCAGAACTGTACTTGTCTGATGGCAAGGTTATTGTGCCACGTCTCAAGCCAGATGTTGACAGAAACCATCGACTGAACTCTGGCCGCCGTCCCATTCTGGTGGACTTGGATCCGAGACAGGAGACTCTGACCCTTGGATACGATGACGATGAGCCTTTCTTCAGGTTCCACGAGGAGCGCTTTGTTCCCCTAGCTGTTGATGAGTCCTTTGTGACGGTCCAGGTGGAGTACTCTCTTGCAAAAGCTCTCAGAATTGGCCAGCTGGGCTACTTCTATCTCATTCAGGGCAGGGTTGCTGGATTCGACGGCACGGTGGTCGCCCTCTCAGAGACCAGCGCTTCCTCTGTTCAAGTCCCTTCCCACCGACTTGTTACCATCACAAACAAGGCGGATCTGACTATGTCTGTGCTTCCAGCCATCTCAGGGGACCTCGTCGCACAGACCCTGCTATCGGACCTCGCCGCAGGTTCATCCGTACTTATCTTTGAGCCTCCCATGCTCTATGTCGAGGCTCTGAGCCATCAAGCAGCGGCTGCTGATATCCCACTCATCTTCGTATCCACCAAGGCCGCTCCGAAATTGGGCGGTATCCGCTGGATTCAACTGCACGAAAAAGAGACGCAGCGCAACCTTCGCCAGAAGCTGCCGCTAAACGCTTCAGTATTTTATGATTTGGCTCCCGAAAAGAGCCCGGCAAGCCTGAACCACAGGCTAGCCAGATGTCTTCCGTCTAGCTGCTCTATTCGCCGAATTGACCATTTGTTTCAGAACGATGCTACACCGATGTCCGCAGAAAGCGTTCAAAACGCGTCTCAGTGTCTGGTTGAGGCTGTCCGGTTAGCAAACGAGGTCAGCAACGACCACAATGTGTTTATACTCAAGGGTAATGAGATATTGTCTTTGAAACAAGGTCCTGAGATCAATGCGGTTGTCGACTGGAAGTCGGACCAGATCATCCCCAGCCGTATCCGCTCGATGGAGACAGATCGGATCTTCGTCGACAACAAGACGTACCTGCTGATTGGTCTGGCTGGTGATCTGGGCCGTTCTATTGCTCGCTTCATGGTCGAGCGCGGCGCACGCCATGTGGTCCTGTCTAGTCGCTCTCCCAAGATTGACCAACGCTGGATCGACGACATCGCCCTGCTTGGGGGAAGCGTGATGGTTCTACCAATGGATGTTTCCAACGAGGCCTCGGTCGACGAAGGTCTCGCTCGAGTCCGTGCCTCTATGCCACCAATTGCTGGTGTTGCCTTTGGACCGCTGGTCCTCCAGGATGTCATGTTCAAGAACATGGACCTATCGATGCTGGAAATGGTGCTTGCGCCCAAGGTGACGGGCGCTCGTCTGTTGAACGAACGTCTCTCGGACCCTGCTAACCCGCTTGACTTCTTTGTCATGTTCTCATCCTTTGTCATGGTCTCCGGGAATCCTGGACAGGCAGCGTACAGCGCTGCAAATGCATACACGCATGCGCTTGCTCAGCAAAGACGCGCTCGTGGTATGGCTGCATCGACCATCGACATTGGTGCCGTGTTCGGTGTCGGCTTCATTGCGAGAGCTGGCCGTGAACACGAGTATGATGTCGTCAGGTTTATCTTCGATGAGGTCAACGAATGGGAGCTGCATGCACTCTTTGCAGAGGCCGTTGTTGCCGGTCGAAACTGGGAGACCAAGGATGTCGAGGTCATCACCGGTATGCCGTACATGGATCCTGCGAACCATGACCGAATTCCCTACTTTGACGACCCGCGATTTGCTTATTTCAGGCTGTCCGATCGTCACATTAAGGGCGAGGACGCCGCTGGTGCGATCGGATCCGTTAAGGACCAACTTATCAAGGCCGAAACCATGGACGACGTTCGCGCGATCATCATCGACGGCCTCTCCGGCAGGATCCGCGGAGCTTTACAGCTTACTCCTGCTGATGAGCTCAATCTGACAGTGCCTCTGATCGATCAGGGCGTGGATTCCCTCAGTGCCGTTACGGTCGGCTCATGGTTCACTAAGAACCTGAATATAGACATTCCCCTTCTGAAGATCCTTGGTGGTGCCTCTGTCGCTGACCTGGTGGACGAGGCAGTCTCCCGCTTGACACCTGAAGCTATTCCCCTGGCTCATTCTACGTCGTCGGAAGATGCAGCGCAGTCTGTCACTGAGGCGCAGCTTATCCCGCCGTCGTCGACAGTTGGCGCGTCCGACGAATCCACGTTCTCTGTTGATTATGATGGCGTTCCAACGCCTCCATCTCCGCATTCCTGCGATGAGGATGGCTTTGAACGGCAGGTACCACTTTCCATCACGCAAGAGTACGCTTGGAAACAACAACAACTTCCCTTGGATCCGACGACGTTCAACAGCACTATTGGCATACACATGCATGGTCCCCTCAATCACAATCGCCTCGGATGGGCATTCAATCAGGCACTGCAGCGGCACGACGCGTTTCGTACATGTTTCATCGCAGATCCGGACGGCTCAGCGCAACCCACCCAAGCCATTATGCGGTCACCGCGAGCCTACTTTGAAGTCGTCAAGGTTATAGACAAAGCTGCCGCTGAGCAAGGGTTCAAGGATCTCGAAGATTACAAGTACGATTTAGCGAAGGGCGACACCCTGAAGATCGTCGTCTTCCACTGGTCACCCACGGATCACCTTCTTATCATTGCTTACCATCGCCTTGTCGGCGATGGCTGGACCACTGAACATCTCTTCGTCGAGGCCGGTCAGTTGTACAGCGGCGTCCCGCTTGAGGCTGCGCCGAGCTACGCCGACTTTGCCATCCGCCAGCGAAAGCAGCTCGAATCCGGCGGCTTCAGTGAGGGCCTCCAATATTGGGGCTGCCTCTTCGAGACCCTTCCCGCAAGACTCCCGGCACTCGACGTCCCCCAGGCCAAGGCGAGTGCCACTCCAACTACCTGGACGGAACACGAGTTGTCCGCACGCCTCAGCAGAATGGCCGCCGTTCGCATCAAAGACCGCAGCCGCAAGCACAAGGTGACGCCTATGCACTACTACCTGGCCTCCTACCTCGTGCTGCTGGCACGCCTCACATCCAGCACAGACGTGGTCATCGGCGTCGCTGACACCAACCGGCCCACGCTCACCGATCAGGCCACTATGGGCTTCTTCGCCAACTTCTTGCCCGTGCGCCTGAACTACAACTCGGACAAGATATTCAATGAGGTTCTCGTAGCTGCCAAGGAACAGATGCGTACGGCGTTGCTGCACAGCGCAGTGCCCTACGGGGCTATGCTCGAGCGGCTCGGCCTGCCAGCCCCATCCGCCGATGACCCGCACTCCCACGCGCCGCTGTTCCAGGCCGTTTTTGACTATAAGCAGGGTCAGGGTGAAAGCGGAAACATTGGTGAGGCGAAGATTATTGATTCGCGCACGCCGCGGGCCGGATCCCCTTATGATATCACCTTGGAGATGAGCGATGACCCTAGCAAGGACCCGTTGATCACGGTCAAGTTGCAGAAGGAGCGGTATGGACCAAAGGATGCTGAGGTGGTCATGGACGCATACCTGTCGATTCTCAGCATCTTTTCGAGGAACCCGGCGCTCAGGGTTGAGGATGGGAGGTTGGATCAGGGGGTCAAATCGAGGGCTTGA | |
| *claD* | | ATGTCTAGGGTACCGTCCAAAGCCCTTCTTTGCTTCCATGGGACTGGCTCGAAGGGAGCCATCTTCAACGTCCAAATGGCGAGAATTTGCTTTCTACTGGGGGAGACGTTCGAGTTCATTTTCCTTGACGGGCCGATGGAATGCGCTGCAGGACCCGGGGTCCTGCCCATGTTCAGCGGCCAAGAGCCATATTACTGTTGGTTTGGTGGCGACGGCACCACTATTGAGAACAGCTTAAAGAGAATCAACGCGTCGGTCAAGCTGACGGTGGATGGATGGCAAACAAATCATCCTGATGCAGAGATCGTCGGAGCGATTGGGTTCTCCGAAGGTGCACTCGCCCTTGCAATGATGCTCTGGCAGCAACAGCACGGCCTAGTGCCCTGGATACCGCAGCTACGATTTGCCGTAATGAGCTGCTGTTTCTTTCCCAACGAGGCTTCCTTGTGGCTGAACGCAAGAGCCCAGGCTTATGGCCTGAGCAAAGCCTACATTAACGTTCCAACGCTTCACATACACGGCAACAGAGACTTTTGTCTTGGCCGCGCACGAAAGCTAGTGAGAAACCACTATCGGTCGAACTTTGCCAATGTCATTCAAACGGAGACCGCCCATCATTTGCCAACAAGGAAAGACGAGGTGGGGGAGGTGGTGAAGCATATCCTGAGACTGTCGGAAATCTCAGCAATTGACGCGCCAAATTGA | |
| *claC* | ATGGGATCGGTTGATACTGCGGCCCTTGTGCCTCCAACAACTCAGCGTGCGGTGATCGTGGAGGCTGGAGATCGGGTGCGCGTACGAGACGATGTACCTTTGCCTACGCTTGAAAACAACCAGTTCTTGATCCGAACGGAAGCCGTGGCTGTCAATCCCAGCGACACCAAAATGCGCGGCGCCTTCGTGACTCCTGGTGGTGTTCTAGGCACTGATTATGCCGGAACCGTCGTCGCTTGTGGGCCTGAGGTCACAGAGGTAGAGGTTGGCGACAGAGTGTGCGGAGCGCAGCATGCCATGAACGCTAACACACCTCATCGTGGATCATTCGGAGAATACAACATTTCTGCTGGCAAGGTCTGGTTGAAGCTACCTGCGTCCGTCAGCACTGAAGGCGGTGCTACGTTTGGTGCCGGTATCAGCACTGCAGGGCTGGCTTTGAAGCTTCTAGGGCTGCCTCTGCCCGATGCTCCAGTTCAGAAGCCTGCATACATTCTTGTGAACGGCGGCAGTACAGCGACCGCAACCATTGCTATACAGCTGCTGAGGCTCGCAAATATGATCCCAATAGCAACCTGTTCGCCAAAAAATTCAGACCAAGTCAAAGCCTACGGTGTCGAAGCGACCTTTGACTATCGCGACCCGGACTGCGCCACTAAGATTAAAGCCTACACGAAAAACAACCTGCGCTACGCTCTCGACTGCATCACGACAGCGGAATCCACCAGCCTCTGCTTCGCCGCCCTTGGTCGCGCCGGCGGCAAGTACGTTTCCCTCGACCCGTTTGCGCAGCACGCCGCCACGCGCGCCACCGTGAAGACCGACTGGGTGCTTGGCCCATCCATCTTCGGTGATGGGTCTACCTGGCCTAATCCATACGGTCGACCGCCGAGTGAGGAGATTCGTGCCTATGGCGAGAAGCTGTGGGCGGTGGCCCAGAAGTTGGTGGATGATGGTAAACTGCGACATCATCCCGTGCGGATCCTTGACGGTGGGCTCGACCAGGTGCTGGTGGGTATGGAGTTAGTGAGGAGTGGCAAGCTTTCAGGGGAAAAATGGGTGGTTCGTCTAGTCAGCTAG | | |
|  |  |  |  |

## Table S4. Structural characterization of clavaic acid (1).

| No. | *δ*_H_ (mult., *J* in Hz) | *δ*_C_, types | HMBC | COSY | NOESY |
| --- | --- | --- | --- | --- | --- |
| 1 | 0.93, m | 48.4, CH | 146.0, 133.2, 50.8, 48.1, 44.2, 43.2, 23.3 | 2.42, 1.83, 1.39 | 1.66, 0.97, 0.77 |
| 2 | 1.39, m | 37.8, CH | 48.1, 23.2 | 1.66, 0.96, 0.93, 0.77 | 1.83 |
| 3α | 1.66, ddd (13.3, 3.0, 3.0) | 48.1, CH_2_ | 48.4, 43.2, 37.8, 33.8, 22.9 | 1.66, 1.54, 1.39 | 1.66 |
| 3β | 0.77, overlap |  | 48.4, 43.2, 33.8 | 1.54, 1.39, 0.77 | 0.93, 0.96 |
| 4 | 1.54, ddd (15.1, 7.0, 3.7) | 33.8, CH | 22.9 | 0.90, 0.77 | 1.83 |
| 5α | 1.74, ddd (12.8, 3.0, 3.0) | 43.2, CH_2_ | 133.2, 48.1, 33.8 | 1.83, 1.54, 0.77 | 0.77, 1.54 |
| 5β | 0.77, overlap |  | 133.2, 48.1, 33.8, 22.9 | 1.83, 1.74, 1.54 | 5.44, 1.74 |
| 6 | 1.83, dddd (12.7, 10.2, 3.0, 2.7) | 44.2, CH | 133.4, 133.2 | 5.44, 1.83, 0.93, 0.77 | 5.44, 2.42, 1.54, 1.39 |
| 7 | 5.44, ddd (9.6, 4.4, 2.7) | 133.2, CH | 133.4, 48.4, 44.2, 43.2, 38.4 | 5.58, 1.83 | 1.83, 1.74 |
| 8 | 5.58, ddd (9.6, 2.0, 2.0) | 133.4, CH | 133.2, 50.8, 44.2, 38.4 | 5.44, 2.18 | 2.18, 0.97 |
| 9 | 2.18, overlap | 38.4, CH | 50.8, 48.4, 16.9 | 5.58, 2.42, 0.97 | 2.42, 0.97 |
| 10 | 2.42, dd (9.8, 5.5) | 50.8, CH | 146.0, 133.4, 130.0, 48.4, 44.2, 38.4, 37,8, 16.9 | 6.03, 2.28, 0.93 | 6.18, 2.18, 1.83, 1.40 |
| 11 | 6.03, dd (15.0, 10.5) | 146.0, CH | 141.1, 130.0, 50.0, 48.4, 38.4 | 6.17, 2.42 | 7.57, 0.97 |
| 12 | 6.17, dd (15.0, 10.3) | 130.0, CH | 146.0, 141.1, 126.9, 50.8 | 6.57, 6.03 | 6.48, 2.42, 0.97 |
| 13 | 6.57, dd (14.8, 10.4) | 141.1, CH | 146.0, 140.0, 130.0, 126.9 | 6.48, 6.17 | 7.22, 6.03 |
| 14 | 6.48, dd (14.7, 11.4) | 126.9, CH | 141.1, 140.0, 130.0, 127.6 | 7.22, 6.57 | 6.17, 1.93 |
| 15 | 7.22, d (11.3) | 140.0, CH | 172.3, 141.1, 127.6, 126.9, 12.8 | 6.48 | 6.57 |
| 16 | NA | 127.6, C | NA | NA | NA |
| 17 | NA | 172.3, C | NA | NA | NA |
| 18 | 0.96, d (5.6) | 23.3, CH_3_ | 48.4, 48.1, 37.8 | 1.39 | 1.66 |
| 19 | 0.90, d (6.6) | 22.9, CH_3_ | 48.1, 43.2, 33.8 | 1.54 | 1.54 |
| 20 | 0.97, d (5.6) | 16.9, CH_3_ | 133.4, 50.8, 38.4 | 2.18 | 6.17, 6.03, 5.58, 2.18 |
| 21 | 1.93, s | 12.8, CH_3_ | 172.3, 140.0, 127.6 |  | 6.48 |

HRMS m/z found 315.2330, calculated for C_21_H_31_O_2_ [M+H]^+^ 315.2319

[α]^20^_D_ :23.6 (c 0.2, MeOH)

# 2 Supplementary Figures

Figure S1. Multiple sequence alignment indicates the conserved HRxxxDG motif found in the condensation domain of LovB-like iPKS.

Figure S2. Comparison of homologous gene clusters from representative species. The amino acid sequence identity to *A. clavatus* PKS cluster is shown. Abbreviations: TF: Transcriptional factor; ER: Enoyl reductase; TE: Thioesterase; PKS: polyketide synthases; mPC: mitochondrial phosphate carrier protein; ACT: Acyltransferase; ACD: Acyl-CoA dehydrogenase; ECH: Enoyl-CoA hydratase; PSD: Phosphatidylserine decarboxylase; MFS: MFS transporter; FMO: Flavin monooxygenase.

Figure S3. UV-Vis absorption spectrum and HR-MS spectrum of clavaic acid (1).

Figure S4. Experimental and calculated ECD spectra of clavaic acid (1).

## Figure S5. ^1^H NMR (500 MHz) **of clavaic acid (1)** in CD_3_OD.

## Figure S6. ^13^C NMR (125 MHz) of clavaic acid (1) in CD_3_OD.

## Figure S7. ^1^H-^13^C HSQC NMR (500 MHz) of clavaic acid (1) in CD_3_OD.

## Figure S8. ^1^H-^1^H COSY (500 MHz) NMR of clavaic acid (1) in CD_3_OD.

## Figure S9. ^1^H-^13^C HMBC (500 MHz) of clavaic acid (1) in CD_3_OD.

## Figure S10. ^1^H-^1^H NOESY of clavaic acid (1) in CD_3_OD.
